# Dr. James McFadden Gaston

**Published:** 1903-12

**Authors:** 


Dr. James McFadden Gaston, of Atlanta, died at his home in that city, November 15, 1903, aged 79 years. He graduated from the Medical College of the State of South Carolina in 1846, and became a medical officer of prominence in the Confederate Army during the Civil War. From 1865 to 1883 he resided in Brazil, where he practised among the southerners, who colonised in that country. After his return to the United States he became the head of the department of surgery in the Southern Medical College at Atlanta, holding the place until declining years compelled him to curtail his professional activity. Dr. Gaston was not only a skilful surgeon, but a man of literary accomplishment, and his writings have been quoted widely at home and abroad. He was president of the Southern Surgical and Gynecological Association in 1892, and of the American Academy of Medicine in 1895. He was elected an honorary fellow of the American Association of Obstetricians and Gynecologists in 1896.

